# Comparative Analysis of Single and Combined Antipyretics Using Patient-Generated Health Data: Retrospective Observational Study

**DOI:** 10.2196/21668

**Published:** 2021-05-26

**Authors:** Yu Rang Park, Hyery Kim, Ji Ae Park, Sang Hyun Ahn, Seyun Chang, Jae Won Shin, Myeongchan Kim, Jae-Ho Lee

**Affiliations:** 1 Department of Biomedical Systems Informatics Yonsei University College of Medicine Seoul Republic of Korea; 2 Department of Pediatrics University of Ulsan College of Medicine Asan Medical Center Children’s Hospital Seoul Republic of Korea; 3 Korea Human Resource Development Institute for Health & Welfare Cheongju Republic of Korea; 4 Mobile Doctor Co, Ltd Seoul Republic of Korea; 5 Department of Information Medicine Asan Medical Center College of Medicine, University of Ulsan Seoul Republic of Korea; 6 Department of Emergency Medicine Asan Medical Center College of Medicine, University of Ulsan Seoul Republic of Korea

**Keywords:** combination antipyretics, fever management, patient-generated health data, comparative analysis, mHealth, apps, fever

## Abstract

**Background:**

Fever is one of the most common symptoms in children and is the physiological response of the human immune system to external pathogens. However, effectiveness studies of single and combined antipyretic therapy are relatively few due to lack of data. In this study, we used large-scale patient-generated health data from mobile apps to compare antipyretic affects between single and combination antipyretics.

**Objective:**

We aimed to establish combination patterns of antipyretics and compare antipyretic affects between single and combination antipyretics using large-scale patient-generated health data from mobile apps.

**Methods:**

This study was conducted using medical records of feverish children from July 2015 to June 2017 using the Fever Coach mobile app. In total, 3,584,748 temperature records and 1,076,002 antipyretic records of 104,337 children were analyzed. Antipyretic efficacy was measured by the mean difference in the area under the temperature change curve from baseline for 6 hours, 8 hours, 10 hours, and 12 hours after antipyretic administration in children with a body temperature of ≥38.0 ℃ between single and combination groups.

**Results:**

The single antipyretic and combination groups comprised 152,017 and 54,842 cases, respectively. Acetaminophen was the most commonly used single agent (60,929/152,017, 40.08%), and acetaminophen plus dexibuprofen was the most common combination (28,065/54,842, 51.17%). We observed inappropriate use, including triple combination (1205/206,859, 0.58%) and use under 38 ℃ (11,361/206,859, 5.50%). Combination antipyretic use increased with temperature; 23.82% (33,379/140,160) of cases were given a combination treatment when 38 ℃ ≤ temperature < 39 ℃, while 41.40% (1517/3664) were given a combination treatment when 40 ℃ ≤ temperature. The absolute value of the area under the curve at each hour was significantly higher in the single group than in the combination group; this trend was consistently observed, regardless of the type of antipyretics. In particular, the delta fever during the first 6 hours between the two groups showed the highest difference. The combination showed the lowest delta fever among all cases.

**Conclusions:**

Antipyretics combination patterns were analyzed using large-scale data. Approximately 75% of febrile cases used single antipyretics, mostly acetaminophen, but combination usage became more frequent as temperature increased. However, combination antipyretics did not show definite advantages over single antipyretics in defervescence, regardless of the combination. Single antipyretics are effective in reducing fever and relieving discomfort in febrile children.

## Introduction

Fever is one of the most common symptoms in children and represents a physiologic response of the human immune system against external pathogens. In particular, fever is reported to be the most common cause of children’s emergency room visits, accounting for 26.4% to 37.4% of visits [1,2]. According to studies in Korea, most causes of childhood fever are viral infections such as mild acute upper respiratory infections [1,2]. However, as parents wish to lower their child’s temperature to within a normal range, antipyretics are commonly administered [3]. This fever phobia has caused caregivers to treat fever aggressively with a combination of antipyretics such as acetaminophen (ACE) and ibuprofen (IBU), often in combination. Despite the lack of official recommendations, combination antipyretic therapy with ACE and IBU is commonly used to treat fever that does not respond to monotherapy [4].

Pharmacologic evidence suggests that the combination of ACE and IBU may be well tolerated because both medications have different metabolic pathways that are not affected by each other [5]. Both drugs are well tolerated, with wide therapeutic margins and proper dosing.

However, because each antipyretic has its own adverse effects, the combination of antipyretics should be properly guided based on reference data regarding antipyretic efficacy. Adverse effects caused by ACE and IBU are well known because of their frequent use worldwide. Hepatic disorders are the most common adverse drug reaction of ACE, whereas gastrointestinal tract disorder, kidney injury, hypersensitivity reaction, and, more recently, noticeable cardiovascular events are frequently reported adverse effects of nonsteroidal anti-inflammatory drugs (NSAIDs) such as IBU [6,7]. In addition, drug misdose or overdose is an important public health issue, especially with antipyretics because most antipyretics are administered to children. Misdosing of antipyretics has been reported in approximately 50% of ACE and 25% of IBU administrations [8-10]; among these, 8.4% to 14% and 9.6% to 14% of patients received higher doses, respectively. Infants aged younger than 1 year were more likely to receive an inaccurate dose than older children because caregivers who used inaccurate doses had difficulty in dosing based on their children’s weight [11]. Overdose of ACE is one of the leading causes of acute liver failure in Western countries [10]. In addition, elevated liver transaminases have been described even at recommended doses in children as well as adults [12].

Several randomized controlled trials that have incorporated combination antipyretics have compared the antipyretic effect and toxicity; these studies favored combination therapy for achieving and sustaining an afebrile state [5,10,13-15]. However, the trial limitations included relatively small numbers of patients and a lack of continuously tracked temperatures, especially in the home setting where combination therapy was administered. Thus, concerns regarding whether combination antipyretics have substantial benefit remain.

To address this concern, drug administration and corresponding temperature recordings are required in a large cohort. These data can be obtained through mobile apps where caregivers can record their child’s data in real time. In this study, we used the mobile app Fever Coach (Mobile Doctor) to obtain real-world data to determine the combination antipyretic use pattern and effects administered at home in numerous febrile children in Korea and compared the antipyretic efficacy between single and combination antipyretics.

## Methods

### Design

To compare the fever reduction effect between single and combination antipyretics, we used temperature and antipyretic records from feverish children collected from July 2015 to June 2017 using the Fever Coach app.

We defined the onset of a fever case when the temperature exceeded 38.0 ℃, and the offset of a fever case when the temperature fell below 38.0 ℃ [16,17]. Temperature values were obtained using linear imputation techniques when values were missing between two neighboring actual temperatures. We used this linear imputation technique with the assumption that fever progression would show a linear characteristic between two short measurements.

The duration was defined as the time elapsed between onset and offset. Antipyretic efficacy was measured by the mean difference between the area under the curve (AUC) and the average temperature changes of the single and combination groups. Because both the degree of temperature elevation and the duration of fever affect patients, the AUC (the product of body temperature and duration) has been used as an indicator for total fever exposure in children and was the study end point to determine the effect of drugs including antipyretics [18-20]. The AUC was calculated using the area under the temperature change curve from baseline for 6 hours, 8 hours, 10 hours, and 12 hours after antipyretic administration in children with a body temperature of ≥38.0 ℃. The populations of single and combination groups were then analyzed with two threshold temperatures at the time of first antipyretic administration: >38 or 39 ℃. We compared the mean difference of the negative AUC from onset through 6 hours, 8 hours, 10 hours, and 12 hours and the average temperature changes over time according to the antipyretic administration pattern.

This study was approved by the institutional review board of Asan Medical Center (2018-0179). The need for informed consent was waived by the ethics committee as this study used routinely collected log data that were anonymously managed at all stages, including during data cleaning and statistical analyses. This study was conducted in accordance with the STROBE statement (Multimedia Appendix 1).

### Setting

The app is based on pediatric thermal standards and assesses a child’s condition based on user input to provide guidelines for antipyretic use. After registration, the user may enter various fever-related information including body temperature, other symptoms, dose and time of antipyretic administration, vaccination and antibiotic history, and physician-made diagnosis if the child was seen by a physician for the management of the current illness. Based on these inputs, the app provides instructions for fever control and advises on the timing of seeking medical attention. The service was designed and reviewed by two board-certified family physicians and one board-certified pediatrician [21]. The app supports the effective and accurate control of common fever symptoms and is available as a free download from Google Play and the App Store. As of June 31, 2017, 393,700 users had registered their child with the app.

**Figure 1:**
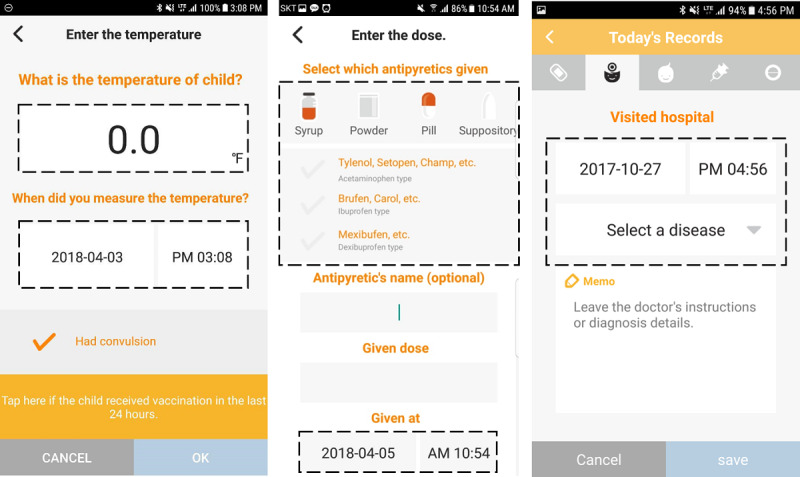
Antipyretic and temperature screens in the Fever Coach app: Enter the temperature (left), Enter the dose (center), Enter the diagnosis (right). The original app was in Korean.

The items in the dashed boxes show the detailed attributes on temperature and antipyretic records and the diagnosis of feverish children (Figure 1). The “Enter the temperature” function records the temperature, time, and experience of febrile convolutions. The “Enter the dose” function records the antipyretic type, ingredients, brand name, doses, and time. The “Today’s records” function records the date and time of the hospital visit, diagnosis, and the doctor’s instruction for the child. The diagnosis includes 21 febrile illnesses that are common in children and diseases that are directly entered.

### Sample and Sampling

**Figure 2:**
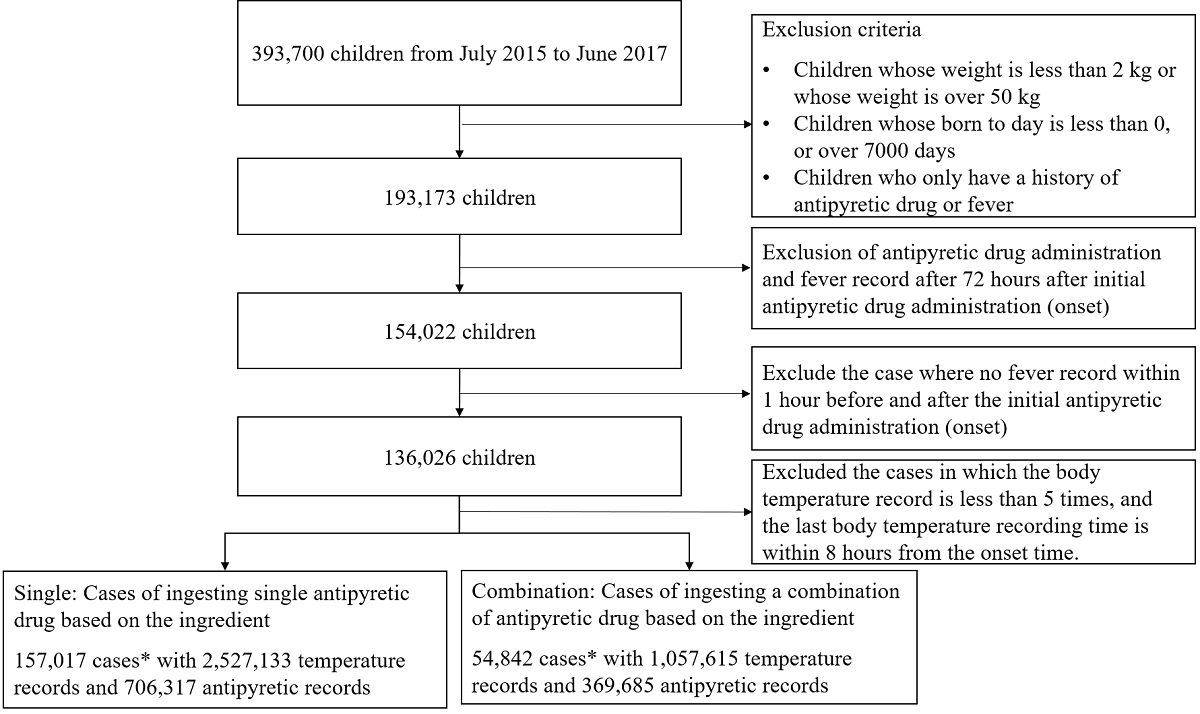
Data collection flow chart. One case refers to a single record for 72 hours after the first antipyretic drug is administered.

We analyzed the log of all users and their children who signed up and logged in to the Fever Coach app more than once from July 2015 to June 2017. Figure 2 shows the target population selection flow of the study. A total of 5,580,762 body temperatures and 1,693,295 antipyretic records were collected on 393,700 children. Among them, we excluded children weighing <2 kg or >50 kg, children <0 days or >7000 days after birth, and children without antipyretic or fever records.

Since a child may experience multiple fevers, one case indicated a single record for 72 hours after the first antipyretic drug was administered. We obtained 494,929 cases with 4,891,960 temperature records and 1,666,346 antipyretic records.

For 72 hours, cases with only one type of antipyretic were defined as a single group (152,019 cases with 2,527,133 temperature and 706,317 antipyretic records), and those including more than one type of antipyretic were recorded as a combination group (54,842 cases with 1,057,615 temperature and 369,685 antipyretic records).

### Statistical Analysis

We compared the demographic and baseline characteristics of children between single and combination groups by means and frequencies using the Student *t* test and chi-square test, respectively. We also compared demographic and body temperature information according to antipyretic administration patterns in single and combination groups by Student *t* test and chi-square test. For the antipyretic effect of single and combination treatment, the AUC values and the fever reduction indicators were analyzed by groups using Student *t* test based on the temperature at the first antipyretic administration (>38 ℃ group, >39 ℃ group). We analyzed the average body temperature change over time by single versus combination and each type of antipyretic administration using a line graph. The effect sizes were calculated using Cohen *d* coefficient interpretation [22]. All reported *P* values were 2-sided, and *P*<.05 was considered significant. Data analyses were conducted using R software (version 4.0.3, R Foundation for Statistical Computing).

## Results

### Overall Characteristics

From July 2015 to June 2017, 3,584,748 temperature records and 1,076,002 antipyretic medication records for 104,337 children were analyzed. Among 206,859 fever cases, the single group comprised 152,017 cases, which encompassed 2,527,133 temperature and 706,317 antipyretic records (Table 1). The combination group comprised 54,842 cases, which encompassed 1,057,615 temperature and 369,685 antipyretic records. When antipyretics were used in combination, they were mostly administered alternatively at 4- to 6-hour intervals (data not shown). As shown in Table 1, most variables were statistically different between the single and combination groups, except the presence of a diagnosis. The proportion of males was slightly higher in the combination group, but no definite male or female predominance was found in either group. Children in the single group were younger (mean 830.49 days) than those in the combination group (mean 848.83 days). The mean temperature at first antipyretic administration was 38.63 ℃ in all children and was higher, at 38.76 ℃, in the combination group and lower, at 38.59 ℃, in the single group, with statistical significance (*P*<.001). The mean maximum temperature in the combination group was higher than that in the single group (single 38.98 ℃ vs combination 39.29 ℃; *P*<.001). However, the mean fever duration was shorter in the single group than in the combination (single 25.08 hours vs combination 28.81 hours; *P*<.001).

When cases were divided by the onset temperature, the most common onset temperatures were between 38 and 39 ℃ in 140,160 cases, followed by those between 39 and 40 ℃ in 51,674 cases. There were 5.49% (11,361/206,859) of cases in whom antipyretics were administered under 38 ℃. In particular, 0.18% (377/206,859) of cases used antipyretics under 37 ℃ (Table 1). The use of combination antipyretics increased with temperature: 23.81% (33,379/140,160) used a combination when the temperature was between 38 and 39 ℃, while 41.40% (1517/3664) used a combination when the temperature was >40 ℃. In contrast, use of single antipyretics decreased from 76.19% (106,781/140,160) to 58.60% (2147/3664) as the temperature increased.

**Table 1:** Descriptive statistics and children’s temperature records according to antipyretic drug administration pattern (single or combination).

Variable		Single (n=152,017)	Combination (n=54,842)	*P* value^a^	Effect size^b^	Total (n=206,859)
Sex, male, n (%)		76,164 (50.10)	28,173 (51.37)	<.001	0.025	104,337 (50.44)
Age (days), mean (SD)		830.49 (534.18)	848.83 (517.23)	<.001	0.035	835.35 (529.80)
Temperature records, n (%)		2,527,133 (70.50)	1,057,615 (29.50)	—^c^	—	3,584,748
Antipyretic records, n (%)		706,317 (65.64)	369,685 (34.36)	—	—	1,076,002
Temperature at first antipyretic,℃ administration,℃, mean (SD)		38.59 (0.53)	38.76 (0.56)	<.001	0.31	38.63 (0.54)
Maximum temperature, ℃, mean (SD)		38.98 (0.67)	39.29 (0.58)	<.001	0.49	39.09 (0.65)
Diagnosis^d^ (ICD-10 CM code), n (%)		1229 (0.81)	457 (0.83)	.58	0.002	1686 (0.82)
Acute upper respiratory infections of multiple and unspecified sites/acute tonsillitis (J06, J03), n (%)		244 (19.985)	105 (22.98)	.16	0.41	349 (20.70)
Acute nasopharyngitis (J00), n (%)		215 (17.49)	76 (16.63)	.68	0.38	291 (17.26)
Influenza and pneumonia (J09-J11), n (%)		193 (15.70)	73 (15.98)	.89	0.37	266 (15.77)
Duration of fever, hours, mean (SD)		25.08 (17.38)	28.81 (16.40)	<.001	0.22	26.02 (17.22)
**Cases according to the onset temperature, n (%)**						
	Temperature < 37 ℃	328 (87.00)	49 (13.00)	<.001	0.03	377
	37 ℃ ≤ Temperature < 38 ℃	8905 (81.07)	2079 (18.93)	<.001	0.09	10,984
	38 ℃ ≤ Temperature < 39 ℃	106,781 (76.19)	33,379 (23.81)	<.001	0.19	140,160
	39 ℃ ≤ Temperature < 40 ℃	33,856 (65.52)	17,818 (34.48)	<.001	0.23	51,674
	Temperature ≥ 40 ℃	2147 (58.60)	1517 (41.40)	<.001	0.09	3664

^a^Student *t* test between the single and combination groups.

^b^Cohen effect size: 0.2, small; 0.5, medium; 0.8, high.

^c^Not applicable.

^d^Diagnosis included within 1 week before and after onset of fever. The 3 most frequently registered diagnoses are listed in order of frequency.

### Antipyretic Drug Administration Pattern

Within the single group, ACE was the most commonly administered (60,929/152,017, 40.08%) single agent, followed by dexibuprofen (DEX) in 36.74% (55,847/152,017) and IBU in 23.18% (35,241/152,017; Multimedia Appendix 2). Within the single group, the mean age was lower in children who were administered ACE than those who were administered IBU or DEX (ACE: 714.27 days, IBU: 902.99 days, DEX: 911.54 days).

In the combination group, the ACE-DEX combination comprised more than half of the total (28,065/54,842, 51.17%), followed by ACE-IBU (22,277/54,842, 40.62%) and IBU-DEX (3295/54,842, 6.01%). Ingesting three antipyretics (ACE-IBU-DEX) accounted for 2.20% (1205/54,842).

In the single group, ACE was the most administered drug when the temperature was between 37 and 38 ℃ in 39.66% (4356/10,984) of cases and between 38 and 39 ℃ in 30.99% (43,442/140,160) of cases. DEX was the second most administered antipyretic agent when the onset temperature was <40 ℃ but was the most administered when the temperature was >40 ℃ in 23.36% (856/3664) of cases. In the combination group, ACE-DEX was the most used combination, regardless of temperature.

ACE as a single agent was not the preferred choice at higher temperatures (43,442/140,160, 30.99%, vs 12,188/51,674, 23.59%, vs 750/3664, 20.47%: 38 to 39 ℃ vs 39 to 40 ℃ vs ≥40 ℃); however, IBU-DEX was used at a similar frequency, regardless of temperature (2032/140,160, 1.45%, vs 1041/51,674, 2.01%, vs 80/3664, 2.18%: 38 to 39 ℃ vs 39 to 40 ℃ vs ≥40 ℃).

### Comparison of Efficacy Between Different Patterns of Antipyretics

For the antipyretic effect of single and combination treatment, the changes of AUC values, as the indicator for total fever exposure, were analyzed by groups based on the temperature at the first antipyretic administration (>38 ℃ group, >39 ℃ group; Table 2). The results according to antipyretic ingredients are summarized in Multimedia Appendix 3. The single group showed significantly higher absolute AUC values than the combination group. In particular, the difference in the AUC during the first 6 hours of fever showed the highest effect size than other times. In the >38 ℃ group, IBU showed the highest AUC at all times, and ACE-IBU showed the highest AUC except for the first 6 hours among combinations. Moreover, the AUC at 12 hours was higher with IBU (–8.00 [SD 5.70] than with ACE-IBU –6.78 [SD 5.21]). Similarly, in the group >39 ℃, IBU had the highest AUC value, and ACE-IBU had the highest value among combinations. Triple combination antipyretics with ACE + IBU + DEX showed the lowest AUC.

**Table 2:** Comparison of the area under the curve between administration patterns.

Time from the onset of fever (hours)			Single, AUC^a^ (SD)		Combination, AUC (SD)	*P* value^b^, AUC (SD)		Effect size^c^, AUC (SD)	Total, AUC (SD)
**Temperature above 38 ℃ at the time of first administration of antipyretic**									
	6	−3.99 (2.87)		−2.86 (2.39)		<.001	0.42		−3.69 (2.79)
	8	−5.21 (3.79)		−4.15 (3.3)		<.001	0.29		−4.92 (3.7)
	10	−6.42 (4.62)		−5.41 (4.21)		<.001	0.22		−6.15 (4.54)
	12	−7.68 (5.47)		−6.64 (5.13)		<.001	0.19		−7.4 (5.4)
**Temperature above 39 ℃ at the time of first antipyretic**									
	6	−5.54 (3.29)		−4.31 (2.64)		<.001	0.41		−5.11 (3.14)
	8	−7.4 (4.33)		−6.23 (3.61)		<.001	0.29		−6.99 (4.13)
	10	−9.31 (5.27)		−8.11 (4.58)		<.001	0.24		−8.89 (5.07)
	12	−11.33 (6.24)		−10 (5.55)		<.001	0.22		−10.8 6 (6.04)

^b^AUC: area under the curve.

^a^Cohen effect size: 0.2, small; 0.5, medium; 0.8, high.

^c^Student *t* test.

**Figure 3:**
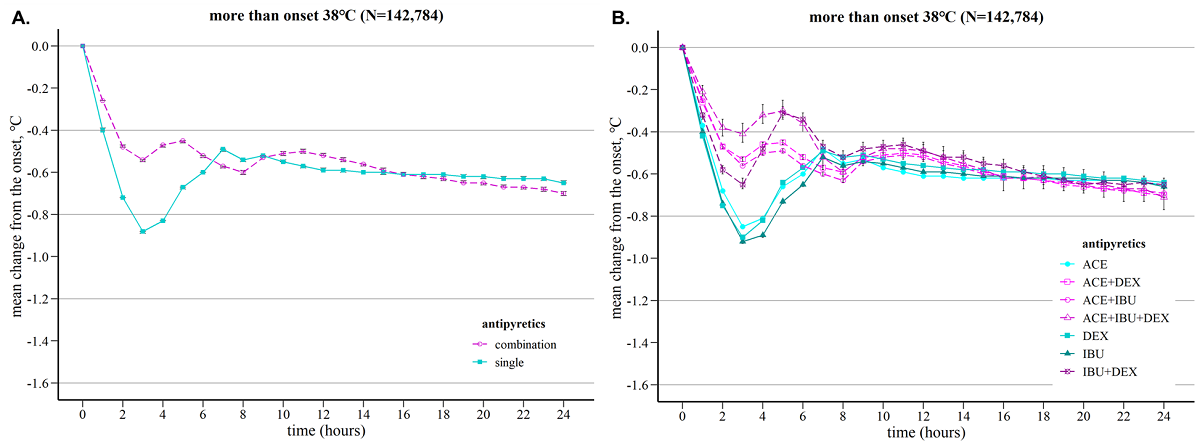
Average temperature changes according to the antipyretic administration pattern for 24 hours in cases where the temperature at the time of antipyretic administration was >38 ℃: (A) comparison of the single and combination groups and (B) comparison of the type of antipyretic agent administered.

The temperature at which the first antipyretic was ingested was defined as the baseline temperature, and the mean difference in temperature for each time over 24 hours was compared between antipyretic administration patterns (Figure 3). As shown in Figure 3A, fever reduction was higher in the single group before 6 hours, and there was little difference in delta fever between the two groups after 6 hours. Comparing the antipyretic agents in the single group, IBU showed the highest fever reduction before 6 hours. However, there was no significant overall difference between agents (Figure 3B). In the combination group, the IBU-DEX combination showed the largest fever difference before 4 hours, but the ACE-IBU combination showed the largest fever difference after 4 hours. After 8 hours, the fever difference between the single and combination groups or antipyretic agents became very small.

## Discussion

### Principal Findings

This is the first and largest cohort study concerning the antipyretic use pattern and antipyretic efficacy between single and combination antipyretics in children using large-scale patient-generated health data (PGHD) on fever, antipyretic agents, and temperature. Most patients used single antipyretics, the most common of which was single ACE. Patients were more likely to use a combination of antipyretics at higher temperatures, and the ACE-DEX combination was the most used. However, fever reduction within 6 hours was significantly better in the single group than in the combination group, as reflected by the AUC analysis. Therefore, a combination of different antipyretics may not have a significant advantage over a single antipyretic. An additional contribution of this study is that we analyzed large-scale real-world PGHD rather than relying on data from a small, clinical-based group; this study used data derived from anyone using a mobile phone and not just from a specific hospital or location, which allowed us to analyze individual data covering a larger area.

### Antipyretics in Use

For fever, ACE is the most prescribed drug, followed by IBU [23]. Both antipyretic agents inhibit prostaglandin synthetase in the hypothalamus, and both are proven to reduce fever [24]. DEX is an NSAID and a pharmacologically effective enantiomer of racemic IBU [25]. DEX can exert identical pharmacological efficacy with smaller doses instead of racemic IBU, which could potentially decrease side effects [25]. In several trials, IBU and DEX showed comparable antipyretic effects and tolerability [25,26]. ACE and IBU or DEX have different modes of action and therefore have been used in combination.

The Fever Coach app is useful to observe antipyretic use patterns in the general pediatric population. In this study, 73.5% of cases used single antipyretics, and most of them used ACE (29.5%). DEX was the second most used antipyretic at 27.0%. ACE was used in younger children and was not frequently administered at higher temperatures. This use pattern might suggest that parents thought ACE was safer but less effective in fever reduction than IBU or DEX. These results also revealed parents’ perceptions of the temperature that required antipyretics. Most cases were administered antipyretics when temperatures were above 38.0 ℃; however, 5.5% used antipyretics under 38 ℃.

Previous studies on parents’ perceptions of fever and antipyretics revealed that many parents were unfamiliar with fever standards [27-29]. In a survey of 105 parents from two emergency rooms in the United States, 81% of parents defined fever as a temperature <38 ℃ [27]. Additionally, 89% reported administering antipyretics to their children even though they seemed comfortable. A survey of 1032 children who visited tertiary hospitals in Turkey revealed that one-third of them had temperatures <37.8 ℃ [29]. These and our results suggest that parents should be educated that temperature does not determine the severity of the disease [1] and antipyretics should not be used to reduce fever but rather to relieve discomfort or pain.

### Patterns of Alternating or Combination Antipyretics

In Korea, surveys on the use or combination of antipyretics are limited [28,30]. Most primary pediatricians recommend alternatively combined rather than simultaneously combined regimens due to concerns regarding additive adverse effects [1,31,32]. Although 4 hours was the most frequent interval in the previous reports, parents also reported alternating therapy every 2 hours, 3 hours, 4 hours, and 6 hours, suggesting that there was no consensus on dosing instructions.

Most cases in the combination group (95%) in our study used an ACE-based combination. ACE-based antipyretics were used in younger children more than other combinations, with an average age of 27.7 months, 28.5 months, and 29.0 months in the ACE-IBU, ACE-DEX, and ACE-IBU-DEX groups, respectively, while the average age in the IBU-DEX group was 30.0 months. Because the average age of ACE cases in the single group was younger than IBU or DEX, the combination pattern in this study might indicate that most parents used ACE first then IBU or DEX in some cases. In this case, patients were more likely to alternatively combine antipyretics at higher temperatures. The ACE-DEX combination was the most administered (12.3% of the combination group) when the temperature was between 38 and 39 ℃, whereas 20.2% administered ACE-DEX when the temperature was >39 ℃. This might be a use pattern reflective of the caregiver’s concerns about fever and the perception that antipyretic combination therapy might be better than monotherapy to reduce fever.

### Effects of Alternating or Combination Antipyretics

Using meta-analysis, IBU was equal or more efficacious in reducing fever than ACE in both pediatric and adult patients and showed similar adverse events [33]. However, previous studies have not uniformly favored combination antipyretics. An Indian study included 89 children using IBU alone or in combination with ACE in which ACE-IBU was more effective than ACE alone, but the effect was <0.5 ℃ [34]. A British study randomized 123 children who received ACE or IBU or both. They reported a temperature difference >1 ℃ between all treatments, but only a 0.35 ℃ difference was found between ACE-IBU and ACE, and 0.25 ℃ between ACE-IBU and IBU [35]. There are currently no clinical guidelines that routinely recommend the combined use of antipyretics and no consistent evidence that combination therapy results in overall improvement in clinical outcomes.

Here, the magnitude of fever reduction within 6 hours, 8 hours, 10 hours, and 12 hours after administration of the first antipyretic agent was significantly better in the single than in the combination group. Patients were more likely to use combination antipyretics at higher temperatures. However, no significant benefit was obtained by adding different antipyretic agents. This trend was consistently observed, regardless of the type of antipyretics used.

NSAIDs have a ceiling effect as analgesics in that there exists a dose beyond which there is no additional effect [36]. Higher doses do not provide additional pain relief but may increase the likelihood of side effects. ACE also demonstrates a ceiling effect in pain relief [37]. However, there is no known ceiling effect in anti-inflammatory doses of NSAIDs or ACE [37]. Thus, the effect of combination antipyretics on fever course remains inconclusive. Based on our results, it is possible that combination antipyretics reduce fever more slowly, indicating more fever exposure during the first several hours administration of the antipyretic agent.

### Adverse Effects of Alternating or Combination Antipyretics

Safety issues are concerning considering the antipyretic combination treatment. In randomized trials in adults with knee pain, drug-related adverse effects were significantly higher in subjects taking combination drugs compared with those taking IBU monotherapy (51% and 42%; *P*=.04) [38]. Moreover, the incidence of diarrhea and hepatotoxicity was significantly higher in the combination therapy group. Additionally, occasional side effects including renal failure [39] and hypothermia were observed [40]. ACE and IBU may cause acute renal failure synergistically as a result of ACE oxidative metabolites accumulating in the renal medulla during renal ischemia, which can be caused by NSAIDs [41,42].

### Limitations

Regarding the limitations of this study, the dataset did not contain information related to adverse effects. In addition, it was difficult to objectively determine the frequency of hypothermia because there were many cases where the body temperature was not entered in the app after the body temperature became normal. However, the concomitant medications and causes of fever would vary between each patient. Thus, it was difficult to assess only antipyretic-related side effects. Although the frequency was very low, there is a risk of serious side effects.

Here, fever was defined as a temperature >38 ℃, regardless of the child’s age. Although fever was originally defined using rectal temperatures, this is not a readily applicable method and infrared tympanic and axillary thermometers are more commonly used [43,44]. However, this dataset did not contain information on the temperature measurement method or site, and it was difficult to analyze all relevant factors, including age and biological factors. Second, a single fever case defined in this study might not be a fever event under the same conditions. Concomitant medication or other general conditions might affect the antipyretic process. Third, since the study was based on patient-generated data, it was dependent on the user entering the data correctly and consistently, and we were unable to collect information on adverse effects. As consistent data could not be collected over time intervals, we used linear interpolation to obtain the body temperature hourly. Moreover, assuming that the change in body temperature over time is linear may have biased the results. Last, there is a possibility of selection bias depending on who used this app first. This app might have been used by caregivers who were either more sensitive or more concerned about fever. Moreover, the combination antipyretics might be more actively selected because of a previous history of fever or febrile convulsions in certain patients. Furthermore, parents with more severely ill children might have a tendency to use combined antipyretics. These limitations could be partially overcome through wearing thermometers and live monitoring equipment in a future study.

### Conclusion

Antipyretic combination patterns were analyzed using real-time PGHD in a large cohort. Approximately 75% of febrile cases used single antipyretics (mostly ACE), but combination use became more frequent as the temperature increased. Single antipyretics showed faster defervescence and reduced the total exposure to fever by duration and temperature. Multiple combined antipyretic administrations did not show definite advantages over single antipyretic use in reducing fever, regardless of the different antipyretics. Moreover, there were also inappropriate uses, such as administering antipyretics at low temperatures or in triple combinations.

Thus, these data suggest that implementation of educational programs and guidelines regarding the proper management of a febrile child are needed, and a mobile app could be a useful platform for this purpose. Single antipyretics are effective in relieving discomfort in febrile children. Combination antipyretics may place children at an increased risk without additional benefit. When educating caregivers, health care providers should minimize fever phobia and emphasize the importance of monitoring the signs and symptoms of a child and improving the child’s comfort in addition to the appropriate dosing of antipyretics.

## Appendix

Multimedia Appendix 1 STROBE Statement for manuscript.

Multimedia Appendix 2 Descriptive statistics and children’s temperature records according to the antipyretic drug administration pattern and antipyretic ingredients.

Multimedia Appendix 3 Comparison of the area under the curve between the administration pattern according to antipyretic ingredient.

## Abbreviations

ACE: acetaminophen
AUC: area under the curve
DEX: dexibuprofen
IBU: ibuprofen
NSAID: nonsteroidal anti-inflammatory drug
PGHD: patient-generated health data

## References

[1] Kim JS (2016). Childhood fever management: current practice vs evidence. Child Health Nurs Res.

[2] Kwak BG, Jang HO (2006). Clinical analysis of febrile infants and children presenting to the pediatric emergency department. Korean J Pediatr.

[3] Sullivan JE, Farrar HC, Section on Clinical Pharmacology and Therapeutics (2011). Fever and antipyretic use in children. Pediatrics.

[4] Mayoral CE, Marino RV, Rosenfeld W, Greensher J (2000). Alternating antipyretics: is this an alternative?. Pediatrics.

[5] Paul IM, Sturgis SA, Yang C, Engle L, Watts H, Berlin CM (2010). Efficacy of standard doses of Ibuprofen alone, alternating, and combined with acetaminophen for the treatment of febrile children. Clin Ther.

[6] Manthripragada AD, Zhou EH, Budnitz DS, Lovegrove MC, Willy ME (2011). Characterization of acetaminophen overdose-related emergency department visits and hospitalizations in the United States. Pharmacoepidemiol Drug Saf.

[7] Nagai J, Uesawa Y, Shimamura R, Kagaya H (2017). Characterization of the adverse effects induced by acetaminophen and nonsteroidal anti-inflammatory drugs based on the analysis of the Japanese adverse drug event report database. Clin J Pain.

[8] Arıkan Z, Tekşam Ö, Kara A, Kale G (2012). Determining causes and frequency of misdosing of antipyretics in patients presenting with fever to pediatric emergency. Turkish Arch Pediatr.

[9] Li SF, Lacher B, Crain EF (2000). Acetaminophen and ibuprofen dosing by parents. Pediatr Emerg Care.

[10] Rodieux F, Piguet V, Desmeules J, Samer CF (2019). Safety issues of pharmacological acute pain treatment in children. Clin Pharmacol Ther.

[11] Wu A Minimizing medication errors in pediatric patients. US Pharm.

[12] Blieden M, Paramore LC, Shah D, Ben-Joseph R (2014). A perspective on the epidemiology of acetaminophen exposure and toxicity in the United States. Expert Rev Clin Pharmacol.

[13] Nabulsi MM, Tamim H, Mahfoud Z, Itani M, Sabra R, Chamseddine F, Mikati M (2006). Alternating ibuprofen and acetaminophen in the treatment of febrile children: a pilot study [ISRCTN30487061]. BMC Med.

[14] Kramer LC, Richards PA, Thompson AM, Harper DP, Fairchok MP (2008). Alternating antipyretics: antipyretic efficacy of acetaminophen versus acetaminophen alternated with ibuprofen in children. Clin Pediatr (Phila).

[15] Hay AD, Costelloe C, Redmond NM, Montgomery AA, Fletcher M, Hollinghurst S, Peters TJ (2008). Paracetamol plus ibuprofen for the treatment of fever in children (PITCH): randomised controlled trial. BMJ.

[16] Craig JV, Lancaster GA, Taylor S, Williamson PR, Smyth RL (2002). Infrared ear thermometry compared with rectal thermometry in children: a systematic review. Lancet.

[17] Davis T (2013). NICE guideline: feverish illness in children—assessment and initial management in children younger than 5 years. Arch Dis Child Educ Pract Ed.

[18] Low JG, Sung C, Wijaya L, Wei Y, Rathore APS, Watanabe S, Tan BH, Toh L, Chua LT, Hou Y, Chow A, Howe S, Chan WK, Tan KH, Chung JS, Cherng BP, Lye DC, Tambayah PA, Ng LC, Connolly J, Hibberd ML, Leo YS, Cheung YB, Ooi EE, Vasudevan SG (2014). Efficacy and safety of celgosivir in patients with dengue fever (CELADEN): a phase 1b, randomised, double-blind, placebo-controlled, proof-of-concept trial. Lancet Infect Dis.

[19] Morris PE, Promes JT, Guntupalli KK, Wright PE, Arons MM (2010). A multi-center, randomized, double-blind, parallel, placebo-controlled trial to evaluate the efficacy, safety, and pharmacokinetics of intravenous ibuprofen for the treatment of fever in critically ill and non-critically ill adults. Crit Care.

[20] Mullins ME, Empey M, Jaramillo D, Sosa S, Human T, Diringer MN (2011). A prospective randomized study to evaluate the antipyretic effect of the combination of acetaminophen and ibuprofen in neurological ICU patients. Neurocrit Care.

[21] Kim M, Yune S, Chang S, Jung Y, Sa SO, Han HW (2019). The Fever Coach mobile app for participatory influenza surveillance in children: usability study. JMIR Mhealth Uhealth.

[22] Cohen J (2013). Statistical Power Analysis for the Behavioral Sciences. 2nd Edition.

[23] Kanabar DJ (2017). A clinical and safety review of paracetamol and ibuprofen in children. Inflammopharmacology.

[24] Hersh EV, Moore PA, Ross GL (2000). Over-the-counter analgesics and antipyretics: a critical assessment. Clin Ther.

[25] Yoon JS, Jeong D, Oh J, Lee KY, Lee HS, Koh YY, Kim JT, Kang JH, Lee JS (2008). The effects and safety of dexibuprofen compared with ibuprofen in febrile children caused by upper respiratory tract infection. Br J Clin Pharmacol.

[26] Rahlfs VW, Stat C (1996). Reevaluation of some double-blind, randomized studies of dexibuprofen (Seractil): a state-of-the-art overview. Studies in patients with lumbar vertebral column syndrome, rheumatoid arthritis, distortion of the ankle joint, gonarthrosis, ankylosing spondylitis, and activated coxarthrosis. J Clin Pharmacol.

[27] Wallenstein MB, Schroeder AR, Hole MK, Ryan C, Fijalkowski N, Alvarez E, Carmichael SL (2013). Fever literacy and fever phobia. Clin Pediatr (Phila).

[28] Choi A, Kim JS (2014). Fever phobia: a survey of children's parents in a pediatric outpatient clinic. Child Health Nurs Res.

[29] Polat M, Kara S, Tezer H, Tapısız A, Derinöz O, Dolgun A (2014). A current analysis of caregivers' approaches to fever and antipyretic usage. J Infect Dev Ctries.

[30] Kwak YH, Kim DK, Jang HY, Kim JJ, Ryu J, Oh SB, Lee EJ, Lee JS, Lee JH, Jung JH, Han SB (2013). Fever phobia in Korean caregivers and its clinical implications. J Korean Med Sci.

[31] Demir F, Sekreter O (2012). Knowledge, attitudes and misconceptions of primary care physicians regarding fever in children: a cross sectional study. Ital J Pediatr.

[32] Wright AD, Liebelt EL (2007). Alternating antipyretics for fever reduction in children: an unfounded practice passed down to parents from pediatricians. Clin Pediatr (Phila).

[33] Pierce CA, Voss B (2010). Efficacy and safety of ibuprofen and acetaminophen in children and adults: a meta-analysis and qualitative review. Ann Pharmacother.

[34] Lal A, Gomber S, Talukdar B (2000). Antipyretic effects of nimesulide, paracetamol and ibuprofen-paracetamol. Indian J Pediatr.

[35] Erlewyn-Lajeunesse MDS, Coppens K, Hunt LP, Chinnick PJ, Davies P, Higginson IM, Benger JR (2006). Randomised controlled trial of combined paracetamol and ibuprofen for fever. Arch Dis Child.

[36] Becker DE (2010). Pain management: part 1: managing acute and postoperative dental pain. Anesth Prog.

[37] Ong CKS, Lirk P, Tan CH, Seymour RA (2007). An evidence-based update on nonsteroidal anti-inflammatory drugs. Clin Med Res.

[38] Doherty M, Hawkey C, Goulder M, Gibb I, Hill N, Aspley S, Reader S (2011). A randomised controlled trial of ibuprofen, paracetamol or a combination tablet of ibuprofen/paracetamol in community-derived people with knee pain. Ann Rheum Dis.

[39] Moghal NE, Hegde S, Eastham KM (2004). Ibuprofen and acute renal failure in a toddler. Arch Dis Child.

[40] Richardson J, Sills J (2004). Hypothermia following fever. Arch Dis Child.

[41] McIntire SC, Rubenstein RC, Gartner JC, Gilboa N, Ellis D (1993). Acute flank pain and reversible renal dysfunction associated with nonsteroidal anti-inflammatory drug use. Pediatrics.

[42] Eguia L, Materson BJ (1997). Acetaminophen-related acute renal failure without fulminant liver failure. Pharmacotherapy.

[43] Craig JV, Lancaster GA, Williamson PR, Smyth RL (2000). Temperature measured at the axilla compared with rectum in children and young people: systematic review. BMJ.

[44] Berksoy E, Bıcılıoğlu Y, Gökalp G, Bal A (2018). Comparison of infrared tympanic, non-contact infrared skin, and axillary thermometer to rectal temperature measurements in a pediatric emergency observation unit. Int J Clin Exp Med.

