# Supplementary material for: Comparative Analysis of Single and Combined Antipyretics Using Patient-Generated Health Data: Retrospective Observational Study
Source: JMIR Mhealth Uhealth. 2021 May 26;9(5):e21668. doi: 10.2196/21668 (PMC8190646; doi:10.2196/21668)
Supplement: Multimedia Appendix 2 [file mhealth_v9i5e21668_app2.docx]

|  | **Single**  **(No. = 152,017)** | | | | **Combination**  **(No. = 54,842)** | | | | |
| --- | --- | --- | --- | --- | --- | --- | --- | --- | --- |
|  | **ACE** | **IBU** | **DEX** | **Total** | **ACE-IBU** | **ACE-DEX** | **IBU-DEX** | **ACE-IBU-DEX** | **Total** |
| Total cases, No. (%) | 60,929 (40.08) | 35,241 (23.18) | 55,847 (36.74) | 152,017 | 22,277 (40.62) | 28,065 (51.17) | 3,295  (6.01) | 1,205  (2.20) | 54,842 |
| Temperature records, No. (%) | 1,031,251(40.81) | 576,502 (22.81) | 919,380 (36.38) | 2,527,133 | 430,540 (40.71) | 541,546 (51.20) | 59,805 (5.65) | 25,724 (2.43) | 1,057,615 |
| Antipyretic records, No. (%) | 274,595 (38.88) | 164,742 (23.32) | 266,980 (37.80) | 706,317 | 149,953 (40.56) | 188,877 (51.09) | 20,345 (5.50) | 10,510 (2.84) | 369,685 |
| Age, days (mean ± SD) | 714.27 ± 495.21 | 902.99 ± 551.47 | 911.54 ± 540.98 | 830.49 ± 534.18 | 830.64 ± 512.82 | 855.54 ± 512.46 | 906.61 ± 564 | 870.86 ± 558.32 | 848.83 ± 517.23 |
| Sex, male, No. (%) | 30,329 (49.78) | 17,837 (50.61) | 27,998 (50.13) | 76,164 (50.10) | 11,459 (51.44) | 14,427 (51.41) | 1,667 (50.59) | 620 (51.45) | 28,173 (51.37) |
| Maximum temperature, ℃, (mean ± SD) | 38.94 ± 0.66 | 39.04 ± 0.65 | 39.06 ± 0.65 | 38.98 ± 0.67 | 39.29 ± 0.58 | 39.3 ± 0.57 | 39.32 ± 0.59 | 39.41 ± 0.51 | 39.29 ± 0.58 |
| Duration time, h, (mean ± SD) | 23.9 ± 17.81 | 26.2 ± 17.11 | 25.68 ± 16.98 | 25.08 ± 17.38 | 29.11 ± 16.42 | 28.52 ± 16.34 | 28.43 ± 16.64 | 30.95 ± 16.69 | 28.81 ± 16.4 |
| ***No. of cases according to the onset temperature*** | | | | | | | | | |
| Temperature < 37℃, No. (%) | 193 (51.19) | 57 (15.12) | 78 (20.69) | 328 (87.00) | 18 (4.77) | 26 (6.90) | 5 (1.33) | 0 (0) | 49 (13.00) |
| 37℃ ≤ temperature < 38℃, No. (%) | 4,356 (39.66) | 1,862 (16.95) | 2,687 (24.46) | 8,905 (81.07) | 876 (7.98) | 1,015 (9.24) | 137 (1.25) | 51 (0.46) | 2,079 (18.93) |
| 38℃ ≤ temperature < 39℃, No. (%) | 43,442 (30.99) | 24,384 (17.40) | 38,955 (27.79) | 106,781 (76.19) | 13,441 (9.59) | 17,212 (12.28) | 2,032 (1.45) | 694 (0.50) | 33,379 (23.81) |
| 39℃ ≤ temperature < 40℃, No. (%) | 12,188 (23.59) | 8,397 (16.25) | 13,271 (25.68) | 33,856 (65.52) | 7,291 (14.11) | 9,071 (17.55) | 1,041 (2.01) | 415 (0.80) | 17,818 (34.48) |
| Temperature ≥ 40℃, No. (%) | 750 (20.47) | 541 (14.77) | 856 (23.36) | 2,147 (58.60) | 651 (17.77) | 741 (20.22) | 80 (2.18) | 45 (1.23) | 1,517 (41.40) |

Multimedia Appendix 2. Descriptive statistics and children’s temperature records according to the antipyretic drug administration pattern and antipyretic ingredients

No: Number of cases, SD: Standard deviation, ACE: Acetaminophen, IBU: Ibuprofen, DEX: Dexibuprofen.
