# Supplementary material for: Comparative Analysis of Single and Combined Antipyretics Using Patient-Generated Health Data: Retrospective Observational Study
Source: JMIR Mhealth Uhealth. 2021 May 26;9(5):e21668. doi: 10.2196/21668 (PMC8190646; doi:10.2196/21668)
Supplement: Multimedia Appendix 3 [file mhealth_v9i5e21668_app3.docx]

Multimedia Appendix 3. Comparison of the area under the curve between the administration pattern according to antipyretic ingredient

| ***Temperature above 38℃ at the time of the first administration of antipyretic (AUC ± SD)*** | | | | | | | | | |
| --- | --- | --- | --- | --- | --- | --- | --- | --- | --- |
| **Time from**  **the onset of fever** | **Single** | | | | **Combination** | | | | |
|  | **ACE**  **(No. = 56,380)** | **IBU**  **(No. = 33,322)** | **DEX**  **(No. = 53,082)** | **Total**  **(No. = 142,784)** | **ACE-IBU**  **(No. = 21,383)** | **ACE-DEX**  **(No. = 27,024)** | **ACE-IBU-DEX**  **(No. = 1,154)** | **IBU-DEX**  **(No. = 3,153)** | **Total**  **(No. = 52,714)** |
| 6 h | −3.83 ± 2.7 | −4.2 ± 3 | −4.04 ± 2.96 | −3.99 ± 2.87 | −2.92 ± 2.42 | −2.83 ± 2.35 | −2.38 ± 2.41 | −2.95 ± 2.47 | −2.86 ± 2.39 |
| 8 h | −5.02 ± 3.59 | −5.48 ± 3.98 | −5.24 ± 3.87 | −5.21 ± 3.79 | −4.26 ± 3.36 | −4.1 ± 3.25 | −3.56 ± 3.33 | −4.04 ± 3.26 | −4.15 ± 3.3 |
| 10 h | −6.23 ± 4.39 | −6.72 ± 4.84 | −6.42 ± 4.69 | −6.42 ± 4.62 | −5.54 ± 4.29 | −5.36 ± 4.15 | −4.8 ± 4.29 | −5.21 ± 4.12 | −5.41 ± 4.21 |
| 12 h | −7.50 ± 5.23 | −8.00 ± 5.70 | −7.66 ± 5.55 | −7.68 ± 5.47 | −6.78 ± 5.21 | −6.59 ± 5.07 | −6.03 ± 5.21 | −6.36 ± 4.97 | −6.64 ± 5.13 |
| ***Temperature above 39 ℃ at the time of first antipyretic (AUC ± SD)*** | | | | | | | | | |
| **Time from**  **the onset of fever** | **ACE**  **(No. = 12,938)** | **IBU**  **(No. = 8,938)** | **DEX**  **(No. = 14,127)** | **Total**  **(No. = 36,003)** | **ACE−IBU**  **(No. =7,942)** | **ACE−DEX**  **(No. =9,812)** | **ACE−IBU−DEX**  **(No. = 460)** | **IBU−DEX**  **(No. = 1,121)** | **Total**  **(No. = 19,335)** |
| 6 h | −5.36 ± 3.14 | −5.77 ± 3.39 | −5.57 ± 3.36 | −5.54 ± 3.29 | −4.37 ± 2.67 | −4.28 ± 2.59 | −3.78 ± 2.76 | −4.4 ± 2.71 | −4.31 ± 2.64 |
| 8 h | −7.19 ± 4.17 | −7.7 ± 4.47 | −7.42 ± 4.37 | −7.4 ± 4.33 | −6.33 ± 3.68 | −6.18 ± 3.54 | −5.66 ± 3.75 | −6.13 ± 3.56 | −6.23 ± 3.61 |
| 10 h | −9.11 ± 5.1 | −9.62 ± 5.43 | −9.31 ± 5.3 | −9.31 ± 5.27 | −8.24 ± 4.67 | −8.05 ± 4.51 | −7.57 ± 4.79 | −7.93 ± 4.5 | −8.11 ± 4.58 |
| 12 h | −11.16 ± 6.08 | −11.65 ± 6.39 | −11.28 ± 6.29 | −11.33 ± 6.24 | −10.14 ± 5.62 | −9.94 ± 5.49 | −9.4 ± 5.77 | −9.71 ± 5.41 | −10 ± 5.55 |

AUC: Area under curve, SD: Standard deviation, No: Number of cases, ACE: Acetaminophen, IBU: Ibuprofen, DEX: Dexibuprofen.
